# Mining the NCBI Influenza Sequence Database: adaptive grouping of BLAST results using precalculated neighbor indexing

**DOI:** 10.1371/currents.RRN1124

**Published:** 2009-11-04

**Authors:** Leonid Zaslavsky, Tatiana Tatusova

**Affiliations:** National Center for Biotechnology Information, National Institutes of Health

## Abstract

The Influenza Virus Resource and other Virus Variation Resources at NCBI provide enhanced visualization web tools for exploratory analysis for influenza sequence data. Despite the improvements in data analysis, the initial data retrieval remains unsophisticated, frequently producing huge and imbalanced datasets due to the large number of identical and nearly-identical sequences in the database.

We propose a data mining algorithm to organize reported sequences into groups based on their relatedness to the query sequence and to each other. The algorithm uses BLAST to find database sequences related to the query. Neighbor lists precalculated from pairwise BLAST alignments between database sequences are used to organize results in groups of nearly-identical and strongly related sequences. We propose to use a non-symmetric dissimilarity measure well crafted for dealing with sequences of different length (fragments).

A balanced and representative data set produced by this tool can be used for further analysis, i.e. multiple sequence alignment and phylogenetic trees. The algorithm is implemented for protein coding sequences and is being integrated with the NCBI Influenza Virus Resource.

## Introduction 

The amount of influenza sequence data is increasing rapidly due to the collaborative genome sequencing efforts [1], [2]. In support of this work, The Influenza Virus Resource [3] has been created at NCBI and is available for public access. This and other Virus Variation Resources at NCBI provide enhanced visualization web tools for exploratory analysis [4], [5], [6] allowing the representation of phylogenetic trees in an aggregated form with a mechanism for refinement. 

Despite the improvements in data analysis, the initial data retrieval remains straightforward in many cases. For example, a database query to NCBI Influenza database is formed as a simple combination of metadata (type of the virus, serotype, segment/proteins, time interval for the date of isolation, etc.) [3] and returns all the sequences that satisfy the query. With the increased number of sequences, it becomes more difficult to operate in this manner: a priori knowledge and experience are required to confirm that all the relevant sequences are included. The dataset extracted in response to the query may be huge and imbalanced due to the large number of identical and nearly-identical sequences in the database (that may differ in metadata though and could not be simply eliminated at the database level). Manual selection of a representative subset is time consuming and difficult. This may create a bottleneck during ongoing pandemic (see [7] for related analysis and references).

In this paper, we describe an algorithm allowing to select the sequences with various degrees of similarity to the query and group the results based on their relatedness to each other. The algorithm uses BLAST scores to find and rate the database sequences related to the query. Pre-calculated neighborhood lists based on pair-wise BLAST hits between the database sequences allow to report the results in groups of identical and nearly identical sequences.

The algorithm is implemented for protein coding sequences and is being integrated with the NCBI Influenza Virus Resource.  

## Method

### Neighbor-relationships between sequences

The standard nucleotide and protein sequence scoring models satisfy conditions (1) - (11) in [8], and the Smith-Waterman local alignment scores satisfy the inequality

\begin{equation*}S^L({\bf x}, {\bf y}) + S^L({\bf y}, {\bf z}) \leq S^L({\bf y},
{\bf y}) + S^L({\bf x}, {\bf z}), \end{equation*}

  

 for any three sequences \begin{equation*}\bf
x\end{equation*}, \begin{equation*}\bf
y\end{equation*}, and \begin{equation*}\bf
z\end{equation*}, and

\begin{equation*}D^L({\bf x}, {\bf y}) = S^L({\bf x}, {\bf x}) + S^L({\bf y}, {\bf
y}) - 2 S^L({\bf x}, {\bf y})
\end{equation*}

         
(1) 

is a metric. Distance (1) provides an adequate measure of sequence closeness if any difference between sequences or absence of data needs to be penalized (two self-scores are calculated over the whole length of the corresponding protein, not over the area of pairwise local alignment). It is used to remove nearly-redundant sequences from a database [8]. Removing near-neighbor redundancy in protein sequence collections is also discussed in [9], and clustered sequence representation is proposed as an alternative in [10]. Though a good measure of similarity between sequences, the alignment metric (1) does not provide an adequate measure of closeness when, for example, a partial sequence is compared to a very similar full-length sequence, and it is not desirable to interpret the absence of the data as a difference. Below we introduce a near-neighbor relationship which seems to be a better measure for cases like that.

Define dissimilarity measure between sequences ${\bf x}$ and ${\bf
y}$:

\begin{equation*}\delta^{L}( {\bf x}, {\bf y} ) = 1 - \frac{ S^L({\bf x}, {\bf y})
}{ S^L({\bf y}, {\bf y}) } \end{equation*}.

(2)    

Sequence ${\bf y}$ is an $\epsilon$-neighbor to sequence ${\bf x}$ if

$\delta^{L}( {\bf x}, {\bf y} ) \leq
\epsilon$,

(3) 

where \begin{equation*}0 < \epsilon <
1\end{equation*}. The maximum value of $\delta^{L}( {\bf x}, {\bf y}
)$ is *1* and it is realized on two unrelated sequences. For identical sequences the value *is 0* . The dissimilarity measure in (2) is non-symmetric: \begin{equation*}\delta^{L}( {\bf x}, {\bf y} ) \neq \delta^{L}( {\bf y}, {\bf x}
).\end{equation*}

That allows a short sequence to be an ε-neighbor to a long sequence, but not vice versa. 

In practice, the highest BLAST bit score between two sequences ("BLAST score"), $S^{BLAST}({\bf x}, {\bf y})$, will be used. BLAST dissimilarity measure

\begin{equation*}\delta^{BLAST}( {\bf x}, {\bf y} ) = 1 - \frac{ S^{BLAST}({\bf x},
{\bf y}) }{ S^{BLAST}({\bf y}, {\bf y}) }
\end{equation*}

      
   (4) 

will be used instead of (2). If BLAST does not find any similarity between \begin{equation*}x\end{equation*} and \begin{equation*}y\end{equation*}, \begin{equation*}\delta^{BLAST}( {\bf x}, {\bf y} ) =
1\end{equation*}. 

Since we are interested in close relationships only and do not consider distant relatives, using fast BLAST algorithm rather than the Smith-Waterman alignment seems reasonable in this context.  

### Creating display groups

A complete list of all reported BLAST hits to a given query sequence may be difficult and sometimes impractical to use due to the swarms of identical and nearly-identical sequences in the search set. The resulting list may be either too long or incomplete depending on the selected threshold. It will be useful to  identify redundant and nearly redundant sequences and produce a near-redundant group report rather than a raw list of all BLAST hits. In our calculations, both relatedness to the query sequence and  pairwise similarity of the database sequences are taken into account.

An important requirement to the algorithm is to allow either query or subject sequence to be incomplete. For example, comparing a coding sequence of HA1 domain of hemaglutinin with complete dataset[11.

Suppose that the subject sequences \begin{equation*}{\bf
x}_0\end{equation*}, \begin{equation*}{\bf x}_1\end{equation*}, ..., \begin{equation*}{\bf
x}_N\end{equation*} are ordered by score to the query sequence \begin{equation*}\bf Q\end{equation*}:

\begin{equation*}S({\bf x}_0, {\bf Q}) \geq S({\bf x}_1, {\bf Q}) \geq ... \geq S({\bf x}_N, {\bf
Q}).\end{equation*}

**Iterations.** During the steps of the algorithm we select the sequences from the ordered list Θ. Initially, set \begin{equation*}\Theta=\{1, 2, ...,
N\}\end{equation*}. At each step we select an anchor sequence \begin{equation*}{\bf
x}_k\end{equation*}. The anchor sequence and all its ε-neighbors form group Λ_κ_ are removed from the list Θ. The procedure is applied until the list Θ becomes empty. 

**Selecting an anchor sequence.** An anchor sequence is selected as follows. A top-score sequence \begin{equation*}{\bf x}_
i\end{equation*} is considered first (e.g., \begin{equation*}i\end{equation*}  is the first in the  list Θ) , and the anchor sequence \begin{equation*}{\bf x}_k\end{equation*} is selected among the sequences in Θ if it is an ε-neighbor to \begin{equation*}{\bf x}_ i\end{equation*} . Specifically, all sequences \begin{equation*}{\bf
x}_{k^{'}}\end{equation*} in the list Θ are selected if  \begin{equation*}{\bf
x}_i\end{equation*} is an ε-neighbor and satisfy the criterion

\begin{equation*}S({\bf x}_{k^{'}}, {\bf Q}) \geq S({\bf x}_{k}, {\bf Q}) - \gamma
S({\bf Q}, {\bf Q})\end{equation*},

(5) 

where \begin{equation*}0 < \gamma \leq
\epsilon\end{equation*}, are considered. The sequence \begin{equation*}{\bf
x}_k\end{equation*} of a  maximum length is selected  as an anchor (see Figure 1). 

**Figure:**
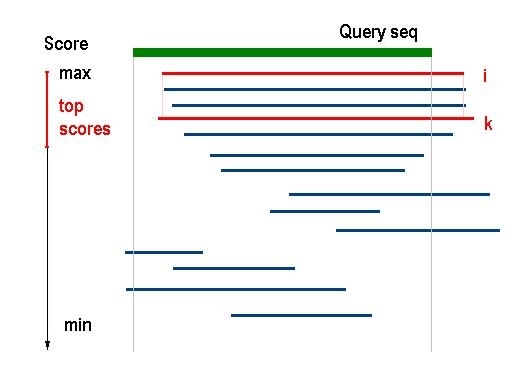
**Fig. 1.** Selecting an anchor sequence.

**Forming a group. **The anchor sequence \begin{equation*}{\bf
x}_k\end{equation*} is selected, then group $\Lambda_k$ is built: all $\epsilon$-neighbors to sequence ${\bf x}_k$ including sequence \begin{equation*}{\bf
x}_k\end{equation*} itself are placed in group $\Lambda_k$ and removed from the list $\Theta$. The iterations are performed until the list $\Theta$ is empty.

**ALGORITHM. Creating display groups.**

**    Sort reported sequences by score:
**       

\begin{equation*}S({\bf x}_0, {\bf Q}) \geq S({\bf x}_1, {\bf Q}) \geq ... \geq S({\bf x}_N, {\bf
Q}).\end{equation*}

  Set    \begin{equation*}\Theta=\{1, 2, ...,
N\}\end{equation*}.

  While ( \begin{equation*}\Theta \neq
\emptyset\end{equation*} ){   

  i. Take the first element \begin{equation*}\bf
i\end{equation*} in the list Θ and consider the list         \begin{equation*}\Pi_i= \{k^{'} \in
\Theta| \delta({\bf x}_{k^{'}}, {\bf x}_i) \leq \epsilon \;\; & \;\; S({\bf x}_{k^{'}}, {\bf
Q}) \geq S({\bf x}_{i}, {\bf Q}) - \gamma S({\bf Q}, {\bf Q})
\}\end{equation*}.

      ii. Find the anchor sequence \begin{equation*}{\bf x}_k = \arg \max \{\;l({\bf x}_{k^\prime} ) \; | \; {\bf
x}_{k^\prime} \in \Pi_i \}\end{equation*}.

      iii. Create group \begin{equation*}\Lambda_k = \{{\bf
x}_j \in \Theta | \delta({\bf x_j, x_k) \leq
\epsilon}\}\end{equation*}.

      iv. Reset \begin{equation*}\Theta \leftarrow
\Theta \backslash \Lambda_k\end{equation*}.

**  } **

**Regularization.** Taking the longest possible sequence \begin{equation*}{\bf
x}_k\end{equation*}  as an anchor instead of sequence \begin{equation*}{\bf x}_
i\end{equation*} allows to create big and stable groups around anchors and avoid fracturing.

**Filtering low-score and low-overlap sequences.** After the groups Λ_κ_ are created, the members of the group that have low-overlap with the query sequence or have much lower score to the query sequence than the anchor, could be filtered out. 

**Indexing.** Steps (i) and (iii) of the Algorithm operate with the sets of ε-neighbors for a sequence. These lists are pre-calculated for the database for preselected values of \begin{equation*}\epsilon
\end{equation*}. As a result, only BLAST scores to the query sequence are calculated on-the-fly.  

#### Showing display groups 

 Each group Λ_κ_ can be displayed at various levels of detail: an anchor sequence and a number of sequences in the group (default) with or without aggregated metadata; groups of identical sequences; and individual sequences. In an aggregated form a group of sequences is shown as a row in the table containing aggregated metadata. For Influenza sequences, it may be score range, identity range, serotype(s), length range, country/countries.).   

## Results

To demonstrate the work of the algorithm, we performed a search for the protein coding sequence of hemagglutinin [Influenza A virus (A/New York/3223/2009(H1N1))], coding for the protein with accession number ACR40288 (all data as of September 20, 2009). The request interface is shown in Figure 2 (Protein accession number [3] [12] or protein coding sequence in either text or FASTA formats [13], [14] can be entered). 

**Figure:**
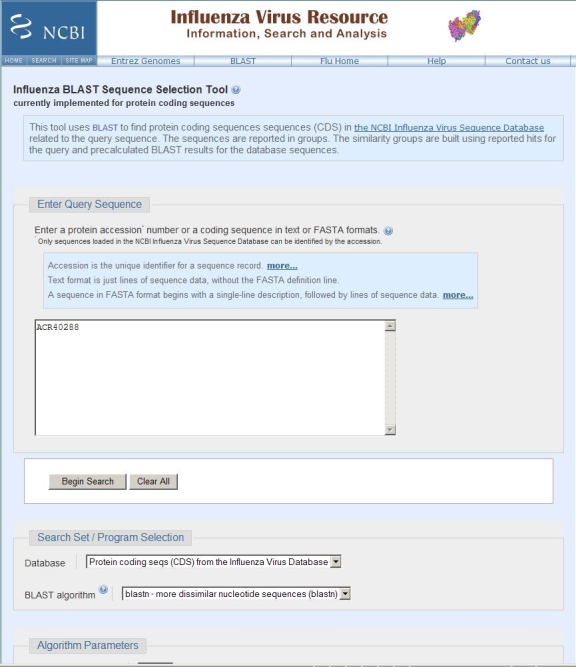
**Figure 2.** Query Interface. 

The results are shown in Figure 3. First group contains 943 near-redundant H1N1 2009 hemagglutinin  protein coding sequences with identity to the query sequence varying from 100% to 99%. The score to the query sequence is varying from 3063 to 1269 due to the sequence length variation from 1701 - 708. While complete sequences in the group contain enire HA coding sequences, partial sequences cover coding sequences of either HA1 or HA2 regions (low-coverage filtering was not turn on in this test). 

Two levels of resolution are shown in Figure 2: table 

**Figure:**
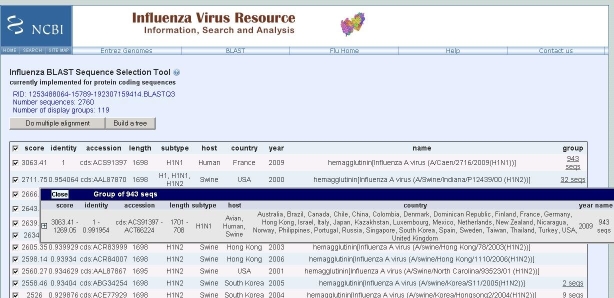
**Figure 3.** Displaying the search results. 

## Discussion

The algorithm presented allows the adaptive creation of display groups, taking into account both relatedness of the reported sequences to the query and their similarity to each other. The groups are built in an efficient manner using pre-calculated ε-neighbors for each sequences. Moreover, additional filtering can be performed, allowing exclusion of members of each group that are less relevant to the query than top members. A balanced and representative set of sequences selected using this tool can be passed to the analysis tools within the NCBI Influenza Virus Resource for calculating multiple sequence alignment and trees [3]. We plan to extend the technology to processing other viruses in the NCBI Virus Variation Resources [4].  

 The developed technology is based on the non-symmetric definition of dissimilarity in (2) and (3).  Measuring dissimilarity in this way is adequate when the absence of the data is considered a probable match (a short sequence is very close to a longer one over the whole length of the former). In some situations, such assumption would not be valid, and a more strict similarity measure, such as the alignment metric (1) should be used [8].

We plan to release a production version of the tool on the web site http://www.ncbi.nlm.nih.gov/genomes/FLU/SmartBLAST.  

## Acknowledgements

The authors are thankful to David J. Lipman, Stephen Altschul, Yiming Bao, James R. Brister, Stacy Ciufo,  William Klimke, Tom Madden, Kathleen O'Neill and Sergey Resenchuk for productive discussions. We appreciate work of Sergey Resenchuk on supporting a BLAST database for influenza virus protein coding sequences.

## Funding Information

This research was supported by the Intramural Research Program of the NIH, National Library of Medicine.

## Competing interests

The authors have declared that no competing interests exist.
